# Serum Zinc Concentration and Sarcopenia: A Close Linkage in Chronic Liver Diseases

**DOI:** 10.3390/jcm8030336

**Published:** 2019-03-11

**Authors:** Hiroki Nishikawa, Hirayuki Enomoto, Kazunori Yoh, Yoshinori Iwata, Yoshiyuki Sakai, Kyohei Kishino, Naoto Ikeda, Tomoyuki Takashima, Nobuhiro Aizawa, Ryo Takata, Kunihiro Hasegawa, Noriko Ishii, Yukihisa Yuri, Takashi Nishimura, Hiroko Iijima, Shuhei Nishiguchi

**Affiliations:** Division of Hepatobiliary and Pancreatic Disease, Department of Internal Medicine, Hyogo College of Medicine, Nishinomiya 663-8501, Hyogo, Japan; enomoto@hyo-med.ac.jp (H.E.); mm2wintwin@ybb.ne.jp (K.Y.); yo-iwata@hyo-med.ac.jp (Y.I.); sakai429@hyo-med.ac.jp (Y.S.); hcm.kyohei@gmail.com (K.K.); nikeneko@hyo-med.ac.jp (N.I.); tomo0204@yahoo.co.jp (T.T.); nobu23hiro@yahoo.co.jp (N.A.); chano_chano_rt@yahoo.co.jp (R.T.); hiro.red1230@gmail.com (K.H.); ishinori1985@yahoo.co.jp (N.I.); gyma27ijo04td@gmail.com (Y.Y.); tk-nishimura@hyo-med.ac.jp (T.N.); hiroko-i@hyo-med.ac.jp (H.I.); nishiguc@hyo-med.ac.jp (S.N.)

**Keywords:** chronic liver disease, zinc, sarcopenia, pre-sarcopenia, liver cirrhosis

## Abstract

We sought to investigate the influence of serum zinc (Zn) concentration on sarcopenia in chronic liver diseases (CLDs, *n* = 372, median age = 65 years, 147 liver cirrhosis (LC) cases (39.5%)). Sarcopenia was defined by low grip strength and low skeletal muscle mass. Study subjects were divided into the following three groups (High-, Intermediate-, and Low-Zn groups) based on the baseline serum Zn level. The impacts of serum Zn concentration on sarcopenia were examined. The median (interquartile range) serum Zn concentration for all cases was 72.85 (63.7, 81.45) μg/dL. The proportions of sarcopenia in the High-Zn, Intermediate-Zn, and Low-Zn groups were 10.75% (10/93), 11.23% (21/187), and 27.17% (25/92), respectively (*P* = 0.9046 (High vs. Intermediate), *P* = 0.0007 (Intermediate vs. Low), *P* = 0.0044 (High vs. Low), overall *P* value = 0.0009). The median serum Zn concentrations in patients with sarcopenia, pre-sarcopenia, and control were 66.35, 73.1 and 73.8 μg/dL, respectively (*P* = 0.0234 (sarcopenia vs. pre-sarcopenia), *P* = 0.2116 (pre-sarcopenia vs. control), *P* = 0.0002 (sarcopenia vs. control), overall *P* value = 0.0016). In the multivariate analyses of factors linked to the presence of sarcopenia, Low-Zn was an independent predictor for all cases (*P* = 0.0236) and LC cases (*P* = 0.0082). In conclusion, Zn deficiency can be an independent predictor for sarcopenia in patients with CLDs.

## 1. Introduction

Zinc (Zn) is an important trace element that is needed for normal cell development, proliferation, and differentiation, and it is also known to be crucial to ensure an appropriate immunological reaction, such as anti-inflammatory effects, anti-oxidant effects, or autophagy [1,2,3,4]. Zn deficiency can cause a wide spectrum of clinical presentations, including appetite loss, body hair loss, impaired taste and smell, atrophy of testis, cerebral and immune dysfunction, and impairment of drug excretion ability, and they are frequently observed in chronic liver diseases (CLDs) as Zn homeostasis is primarily regulated in the liver [3,5,6,7,8,9]. Albumin synthesis disability can cause Zn deficiency in patients with liver cirrhosis (LC) [3,10,11]. Serum Zn concentrations had the inverse correlation with serum ammonia levels in LC patients [12,13,14]. The degree of Zn deficiency is reported to correlate well with the severity of liver diseases [15]. On the other hand, Zn deficiency-related abnormalities may be covered by Zn supplementation [3,5,6,7,13]. Katayama et al. reported in their randomized controlled trial that Zn supplementation therapy can be safe and effective for treating hyperammonemia in LC patients [13]. However, numerous clinical aspects of Zn deficiency have not yet been elucidated in CLD patients.

Skeletal muscle mass (SkMM) decreases by approximately 1% after the age of 50 years due to the qualitative and quantitative changes in muscle fibers [16]. Sarcopenia is a common syndrome mainly observed in older population and characterized by progressive decline of muscle mass and muscle function resulting in an increased risk of physical disability, decreased quality of life (QOL), and mortality [17,18,19,20,21,22,23]. In CLD patients, sarcopenia can also be observed irrespective of age due to protein-energy malnutrition or other metabolic or hormonal disorders specific to CLDs [18,19,21,23,24,25,26,27,28]. Sarcopenia in CLDs is therefore a serious health problem. Based on these backgrounds, the Japanese Society of Hepatology (JSH) proposed its own criteria for liver disease-related sarcopenia in 2016 [18]. Notably, there is no age restriction for the evaluation of sarcopenia in the JSH guidelines [18]. Numerous evidences for liver disease-related sarcopenia have been accumulated [28,29,30,31]. 

However, as far as we are aware, the association between serum Zn concentration and sarcopenia in CLD patients is largely unknown. There seems therefore to be a pressing need for clarifying these issues. In this study, we sought to investigate the influence of serum Zn concentration on sarcopenia in patients with CLDs. 

## 2. Patients and methods

### 2.1. Patients

A total of 378 CLD patients with data for grip strength (GpS), SkMM using bioimpedance analysis (BIA), and serum Zn concentration were admitted to our institution between November 2013 and August 2018. Because overestimates could occur for the calculation of skeletal muscle mass index (SMI) using BIA in patients with massive ascites, 6 subjects with massive ascites were excluded from the study [19]. Three-hundred and seventy-two patients were consequently included in the current analysis. CLD was determined according to patient medical record, laboratory data, histological findings, and imaging findings. LC diagnosis was also based on histological findings (F4) or imaging findings (presence of varices, deformity of the liver, splenomegaly, etc.). 

### 2.2. Our Classification Based on the Serum Zn Concentration

**Figure 1:**
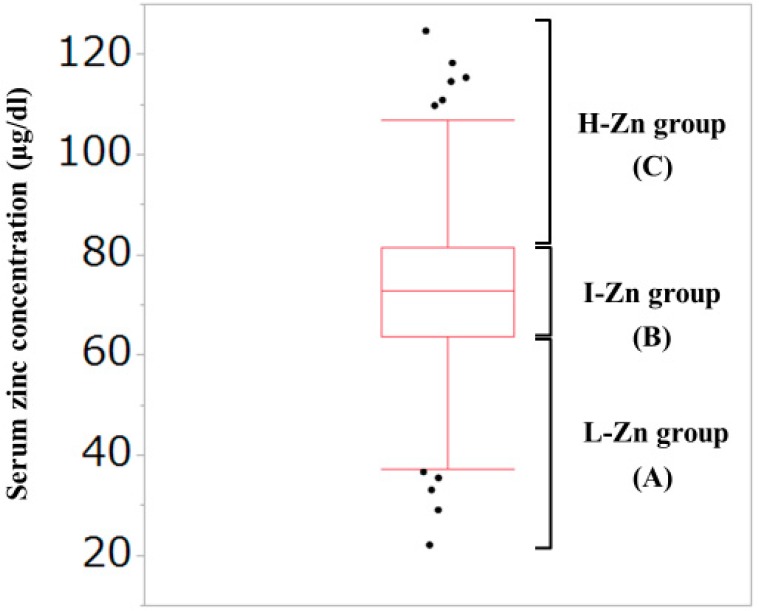
Our classification based on the serum Zn concentration. The median (interquartile range) serum Zn concentration for all cases in the current analysis was 72.85 (63.7, 81.45) μg/dL. (**A**) Low-Zn group; patients with serum Zn concentration <63.7 μg/dL (first quartile). (**B**) Intermediate-Zn group; patients with 63.7 μg/dL <serum Zn concentration <81.45 μg/dL (second or third quartile). (**C**) High-Zn group; patients with serum Zn concentration >81.45 μg/dL (fourth quartile).

As described later, the median (interquartile range (IQR)) serum Zn concentration for all cases in the current analysis was 72.85 (63.7, 81.45) μg/dL. Thus, our study subjects were divided into the following three groups based on the baseline serum Zn level: (A) Low-Zn (L-Zn) group; patients with serum Zn concentration <63.7 μg/dL (first quartile). (B) Intermediate-Zn (I-Zn) group; patients with 63.7 μg/dL ≤serum Zn concentration ≤81.45 μg/dL (second or third quartile). (C) High-Zn (H-Zn) group; patients with serum Zn concentration >81.45 μg/dL (fourth quartile). (Figure 1) The normal range of serum Zn concentration in our institution is from 80 μg/dL to 130 μg/dL. Hypozincemia was thus defined as serum Zn concentration <80 μg/dL. 

### 2.3. Measurement of GpS and SMI for Evaluating Sarcopenia

GpS was measured according to the current Japanese guidelines [18]. SMI was defined as “appendicular SkMM/(height (m))^2^” using BIA. According to the current Japanese guidelines, patients with decreased GpS were defined as those with GpS <26 kg for male and <18 kg for female. Similarly, patients with decreased SkMM were defined as those with SMI <7.0 kg/m^2^ for male and <5.7 kg/m^2^ for female [18]. In males, patients with GpS <26 kg and SMI <7.0 kg/m^2^ were classified as having sarcopenia, those with GS <26 kg and SMI ≤7.0 kg/m^2^ as pre-sarcopenia, those with GS ≤26 kg and SMI <7.0 kg/m^2^ as pre-sarcopenia, and those with GS ≤26 kg and SMI ≤7.0 kg/m^2^ as control. In female, patients with GS <18 kg and SMI <5.7 kg/m^2^ were classified as sarcopenia, those with GS <18 kg and SMI ≤5.7 kg/m^2^ as pre-sarcopenia, those with GS ≤18 kg and SMI <5.7 kg/m^2^ as pre-sarcopenia, and those with GS ≤18 kg and SMI ≤5.7 kg/m^2^ as control.

Firstly, the impacts of serum Zn concentration on sarcopenia were examined for all cases and several subgroups according to the LC status. Secondly, factors associated with the presence of sarcopenia were studied using univariate and multivariate analyses. Correlation between serum Zn concentration and baseline characteristics was also examined. The ethics committee of our hospital acknowledged this study (number: 2296). The protocol in the study strictly observed all regulations of the Declaration of Helsinki. 

### 2.4. Statistical Considerations

As for continuous parameters, Student’s *t* test, Mann-Whitney U test, Pearson’s correlation coefficient *r*, analysis of variance, or Kruskal-Wallis test were employed to assess group difference, as applicable. In categorical parameters, Pearson χ^2^ test was employed to assess group difference, as applicable. Factors with *P* < 0.05 linked to the presence of sarcopenia in the univariate analysis were subjected to the multivariate logistic regression analysis to identify candidate parameters. Baseline factors significantly correlated with serum Zn concentration in the univariate analysis were also subjected to the multivariate logistic regression analysis to identify candidate parameters. Unless otherwise mentioned, data were indicated as median values (IQR). The threshold for statistical significance was set at *P* < 0.05. The JMP 13.2 (SAS Institute Inc., Cary, NC, USA) was employed to carry out statistical analysis.

## 3. Results

### 3.1. Patient Baseline Characteristics

**Figure 2:**
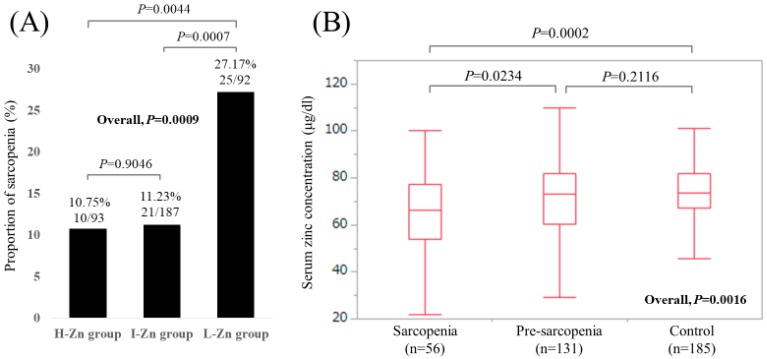
(**A**) The proportions of sarcopenia in the High-Zn, Intermediate-Zn, and Low-Zn groups. (**B**) Boxplots of serum Zn concentration in the High-Zn, Intermediate-Zn, and Low-Zn groups. The median (IQR) serum Zn concentrations in patients with sarcopenia, pre-sarcopenia, and control were 66.35 (54.05, 77.425) μg/dL, 73.1 (60.4, 82.1) μg/dL, and 73.8 (67.2, 81.85) μg/dL, respectively.

Baseline characteristics in our study (*n* = 372, 171 males and 201 females, median age (IQR) = 65 (54, 71.75) years) were shown in Table 1. In terms of liver disease etiology, hepatitis C virus was in the majority (60.2%, 224/372). The median (IQR) serum Zn concentration for all cases was 72.85 (63.7, 81.45) μg/dL. Hypozincemia (<80.0 μg/dL) was identified in 261 patients (70.2%). Sarcopenia was observed in 56 patients (15.1%), while pre-sarcopenia (patients with decreased GpS alone or decreased SkMM alone) was observed in 131 patients (35.2%). There were 93, 187, and 92 patients in the H-Zn, I-Zn, and L-Zn groups, respectively. The proportions of sarcopenia in the H-Zn, I-Zn, and L-Zn groups were 10.75% (10/93), 11.23% (21/187), and 27.17% (25/92), respectively (*P* values: *P* = 0.9046 (H-Zn group vs. I-Zn group), *P* = 0.0007 (I-Zn group vs. L-Zn group), *P* = 0.0044 (H-Zn group vs. L-Zn group), overall *P* value = 0.0009) (Figure 2A). The median (IQR) serum Zn concentrations in patients with sarcopenia, pre-sarcopenia, and control were 66.35 (54.05, 77.425) μg/dL, 73.1 (60.4, 82.1) μg/dL, and 73.8 (67.2, 81.85) μg/dL, respectively (*P* values: *P* = 0.0234 (sarcopenia vs. pre-sarcopenia), *P* = 0.2116 (pre-sarcopenia vs. control), *P* = 0.0002 (sarcopenia vs. control), overall *P* value = 0.0016) (Figure 2B).

### 3.2. Analyses According to the LC Status

**Figure 3:**
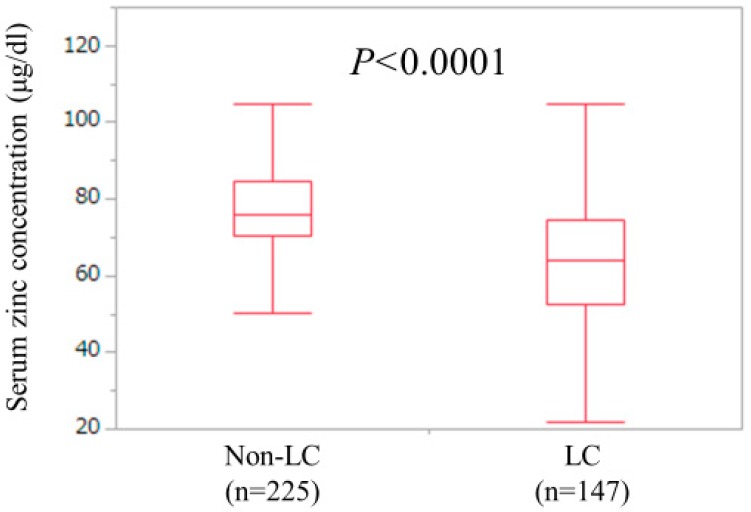
Serum Zn concentrations in LC (*n* = 147) and non-LC (*n* = 225) patients. The median (IQR) serum Zn concentration for LC cases (63.8 (52.7, 74.4) μg/dL) was significantly lower than that for non-LC cases (75.8 (70.45, 84.45) μg/dL) (*P* < 0.0001).

LC and non-LC were identified in 147 patients (39.5%) and 225 patients (60.5%) in the current analysis. The median (IQR) serum Zn concentration for LC cases (63.8 (52.7, 74.4) μg/dL) was significantly lower than that for non-LC cases (75.8 (70.45, 84.45) μg/dL) (*P* < 0.0001) (Figure 3). One-hundred and twenty-four LC patients (84.3%) and 137 non-LC patients (60.9%) had hypozincemia. 

**Figure 4:**
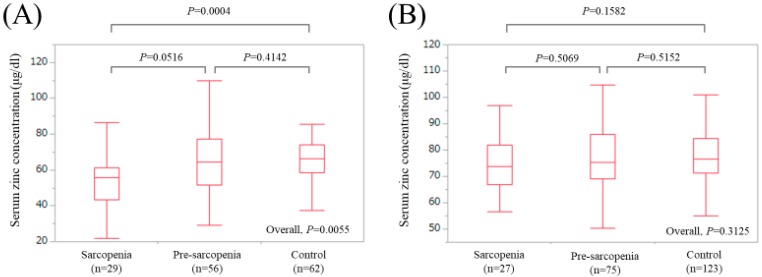
Boxplots of serum Zn concentration in the High-Zn, Intermediate-Zn, and Low-Zn groups in patients with LC (**A**) and non-LC (**B**). In LC patients, the median (IQR) serum Zn concentrations in sarcopenia, pre-sarcopenia, and control were 55.7 (43.15, 61.4) μg/dL, 64.6 (51.55, 77.325) μg/dL, and 66.4 (58.45, 74.175) μg/dL, respectively. In non-LC patients, the median (IQR) serum Zn concentrations in sarcopenia, pre-sarcopenia, and control were 73.9 (66.9, 81.9) μg/dL, 75.4 (69.0, 86.0) μg/dL, and 76.5 (71.2, 84.3) μg/dL, respectively.

In LC patients, there were 29 (19.7%), 56 (38.1%), and 62 (42.2%) patients with sarcopenia, pre-sarcopenia, and control, respectively. The median (IQR) serum Zn concentrations in LC patients with sarcopenia, pre-sarcopenia, and control were 55.7 (43.15, 61.4) μg/dL, 64.6 (51.55, 77.325) μg/dL, and 66.4 (58.45, 74.175) μg/dL, respectively (*P* values: *P* = 0.0516 (sarcopenia vs. pre-sarcopenia), *P* = 0.4142 (pre-sarcopenia vs. control), *P* = 0.0004 (sarcopenia vs. control), overall *P* value = 0.0055) (Figure 4A). 

In non-LC patients, there were 27 (12.0%), 75 (33.3%), and 123 (54.7%) patients with sarcopenia, pre-sarcopenia, and control, respectively. The median (IQR) serum Zn concentrations in non-LC patients with sarcopenia, pre-sarcopenia, and control were 73.9 (66.9, 81.9) μg/dL, 75.4 (69.0, 86.0) μg/dL, and 76.5 (71.2, 84.3) μg/dL, respectively (*P* values: *P* = 0.5069 (sarcopenia vs. pre-sarcopenia), *P* = 0.5152 (pre-sarcopenia vs. control), *P* = 0.1582 (sarcopenia vs. control), overall *P* value = 0.3125) (Figure 4B).

### 3.3. Uni- and Multivariate Analyses of Factors Associated with the Presence of Sarcopenia for All Cases (n = 372)

In all cases, univariate analysis identified nine factors to be significantly associated with the presence of sarcopenia: age (*P* < 0.0001), body mass index, (BMI, *P* < 0.0001), presence of LC (*P* = 0.0416), total bilirubin (*P* = 0.0276), serum albumin (*P* = 0.0008), alkaline phosphatase (ALP, *P* = 0.0143), estimated glomerular filtration rate (eGFR, *P* = 0.0178), serum sodium (*P* = 0.0164), and our classification of serum zinc concentration (*P* = 0.0009) (Table 2). Multivariate analysis for the nine factors showed that age (*P* = 0.0005), BMI (*P* = 0.0003), serum albumin (*P* = 0.0011), and L-Zn group (*P* = 0.0236, I-Zn group as a reference) were found to be significant factors linked to the presence of sarcopenia (Table 3). Hazard ratios (HRs) and 95% confidence intervals (CIs) for these variables were listed in Table 3.

### 3.4. Uni- and Multivariate Analyses of Factors Associated with the Presence of Sarcopenia for LC Cases (n = 147)

In LC-cases, univariate analysis identified four factors to be significantly associated with the presence of sarcopenia: age (*P* = 0.0026), BMI (*P* = 0.0010), serum albumin (*P* = 0.0035), and our classification of serum zinc concentration (*P* = 0.0016) (Table 4). Multivariate analysis for the four factors showed that age (*P* = 0.0363), BMI (*P* = 0.0014), and L-Zn group (*P* = 0.0082, I-Zn group as a reference) were found to be significant factors linked to the presence of sarcopenia (Table 5). HRs and 95% CIs for these variables were listed in Table 5. 

### 3.5. Uni- and Multivariate Analyses of Factors Associated with the Presence of Sarcopenia for Non-LC Cases (n = 225)

In non-LC cases, univariate analysis identified four factors to be significantly associated with the presence of sarcopenia: age (*P* < 0.0001), BMI (*P* = 0.0003), eGFR (*P* = 0.0063), and HbA1c (*P* = 0.0091) (Table 6). Multivariate analysis for the four factors showed that age (*P* = 0.0249) and BMI (*P* = 0.0021) were found to be significant factors linked to the presence of sarcopenia (Table 7). HRs and 95% CIs for these variables were listed in Table 7. 

### 3.6. Correlation between Serum Zinc Concentration and Baseline Characteristics

Correlation coefficients and *P* values between serum zinc concentration and baseline characteristics were listed in Table 8. Serum albumin level had the strongest positive correlation with serum Zn concentration (*r* = 0.654, *P* < 0.0001) followed by branched-chain amino acid to tyrosine ratio (*r* = 0.496, *P* < 0.0001), while FIB-4 index had the strongest negative correlation with serum Zn concentration (*r* = −0.360, *P* < 0.0001) followed by total bilirubin (*r* = −0.355, *P* < 0.0001). Multivariate analysis of factors significantly correlated with serum Zn concentration revealed that only serum albumin level was a significant factor linked to serum Zn concentration (*P* < 0.0001). 

## 4. Discussion 

Chronic liver injury results in the impaired Zn homeostasis and eventually Zn deficiency, and most CLD patients have Zn deficiency [3,4,5,10,13,14,32]. Zn deficiency can present numerous symptoms, which can be associated with decreased QOL and worse clinical outcomes [3,5,33]. However, as mentioned earlier, there is little data currently available regarding relationship between serum Zn concentration and sarcopenia in CLDs. To clarify these issues is clinically of great importance due to the increasing interest for sarcopenia in CLDs these days. To the best of our knowledge, this is the first study elucidating the association between serum Zn level and sarcopenia in CLDs using large cohort (*n* = 372). 

First of all, the validity of our classification of H-Zn, I-Zn, and L-Zn groups needs discussion. As mentioned above, CLD patients have tendency for lower serum Zn levels and only 111 of our analyzed subjects (29.8%) had normal serum Zn value (reference range, 80 μg/dL ≤serum Zn concentration ≤130 μg/dL in our institution) [3,5,7]. Therefore, we classified them into three groups based on quartiles in our cohort for the appropriate analysis and investigated the impact of serum Zn concentration on sarcopenia. In our analysis, the proportion of sarcopenia in the L-Zn group was significantly higher than that in the I-Zn group or the H-Zn group. Additionally, our multivariate analyses revealed that L-Zn was an independent predictor linked to the presence of sarcopenia for all cases and LC cases. These results suggest the clinical significance of serum Zn concentration for sarcopenia in CLD patients, even considering the non-significant relation between serum Zn concentration and sarcopenia in non-LC cases. Zn deficiency induces a number of physiological problems, which can be associated with sarcopenia [34]. In CLD patients, close monitoring for serum Zn concentration may be required for identifying sarcopenic subjects. While patients with pre-sarcopenia had similar serum Zn concentration to those with control for all cases, LC cases, and non-LC cases. Reviewing these results, sarcopenia staging (i.e., distinguishing between sarcopenia and pre-sarcopenia) seems to be an important strategy for CLD patients. A recent study reported that a stepwise increase of the proportion of fallers according to the severity of sarcopenia was observed in older populations, which was in line with our current results [35]. On the other hand, appropriate timing of Zn supplementation therapy should also be considered in CLD patients from the clinicians’ perspective. In addition, whether Zn supplementation therapy in sarcopenic CLD patients can reverse sarcopenia needs to be clarified in future studies. 

In comparison between L-Zn group and H-Zn group, the impact on sarcopenia was not prominent in the multivariate analysis for all cases (HR = 1.672, *P* = 0.3881, H-Zn as a reference). Although the reasons for these are unclear, maintaining serum Zn concentration in a certain range may be essential for avoiding sarcopenia in CLD patients. 

Serum albumin level had the strongest positive correlation with serum Zn concentration (*r* = 0.654, *P* < 0.0001) and it was an independent predictor associated with serum Zn concentration in the multivariate analysis, while FIB-4 index had the strongest negative correlation with serum Zn concentration (*r* = −0.360, *P* < 0.0001) in our data. Because protein synthesis ability reflects serum Zn concentration, these results are not so surprising [3,5,7]. More importantly, age was correlated inversely with serum Zn concentration, although the *r* value was not impressive (*r* = −0.186, *P* = 0.0003). In aging process, a lot of mediators can be associated with inflammation and oxidative stress, and imbalance of Zn homeostasis is a common hallmark of aging [36,37,38]. The high prevalence of hypozincemia in our LC patients (84.3%) may be partly attributed to the higher age of our LC patients (median age = 68.0 years), as well as protein synthesis inability and other metabolic disorders [3,5,7]. On the other hand, clinicians should be aware that even in non-LC patients, a considerable number of patients with hypozincemia are present (60.9% in our non-LC patients).

Both age and BMI were independent predictors for all cases, LC cases, and non-LC cases in our multivariate analyses. CLD patients appears to suffer from sarcopenia based on the following two types of sarcopenia: aging-related sarcopenia (primary sarcopenia) and CLD specific abnormalities-related sarcopenia (secondary sarcopenia) [18]. Our current results may be attributed to the increase of aging CLD patients in our country [39,40,41]. As CLD patients age, the incidence of physical performance limitation will increase and sarcopenia may be an inevitable consequence with aging [42]. While lower BMI was shown to have strong influence on sarcopenia in our CLD patients, the impact of obesity on sarcopenia (sarcopenic obesity) cannot be ignored, since there were only 3 patients (0.8%) with BMI >35 kg/m^2^ in our study subjects [43,44].

We acknowledge several limitations to this study. First, the study was a retrospective single-center observational study without non-CLD patients as a control group. Second, GpS and serum Zn concentration can vary depending on patient daily life activities or dietary habits. Third, patients with massive ascites potentially involved in liver disease-related sarcopenia were excluded because of the lack of reliability in BIA, creating bias. This was the major limitation for the evaluation of muscle mass using BIA compared with using computed tomography or magnetic resonance imaging, however, BIA may be cost-effective. Fourth, data for other trace elements such as calcium, iron, phosphorus, magnesium, and selenium were not included in this study, leading to bias. Fifth, due to the small number of cases with sarcopenic obesity in our study, the relationship between serum Zn concentration and sarcopenic obesity remains unknown. Finally, it was uncertain as to whether sarcopenia caused Zn deficiency or whether Zn deficiency caused sarcopenia in this cross-sectional study. Consequently, caution should be exercised for the interpretation of the current study data and larger prospective studies will be needed to confirm these results. Despite these limitations, our study results denoted that Zn deficiency in CLDs was closely associated with sarcopenia. 

## 5. Conclusions

In conclusion, Zn deficiency can be an independent predictor for sarcopenia in patients with CLDs. 

## Figures and Tables

**Table 1:** Baseline characteristics.

Variables	All Cases (*n* = 372)
Age (years)	65 (54, 71.75)
Gender, male/female	171/201
HBV/HCV/HBV and HCV/NBNC	53/224/8/87
Presence of LC, yes/no	147/225
Body mass index (kg/m^2^)	22.6 (20.4, 25.3)
Total bilirubin (mg/dL)	0.8 (0.6, 1.1)
Serum albumin (g/dL)	4.1 (3.8, 4.5)
Prothrombin time (%)	88.5 (78.3, 96.5)
Platelet count (×10^4^/mm^3^)	16.5 (11.35, 20.7)
Total cholesterol (mg/dL)	179 (151, 206)
AST (IU/L)	28 (21, 42)
ALT (IU/L)	23 (15, 40)
ALP (IU/L)	243 (193, 318.25)
GGT (IU/L)	28 (17, 50)
eGFR (mL/min/1.73 m^2^)	83 (70, 96)
HbA1c (NGSP)	5.6 (5.4, 6.0)
Serum sodium (mmol/L)	140 (139, 142)
Serum zinc concentration (μg/dL)	72.85 (63.7, 81.45)
BTR	5.63 (4.2375, 6.65)
FIB-4 index	2.39 (1.60, 4.07)
APRI	0.52 (0.33, 1.15)

Data are expressed as median value (interquartile range). Abbreviations: HBV, hepatitis B virus; HCV, hepatitis C virus; NBNC, non-B and non-C; LC, liver cirrhosis; AST, aspartate aminotransferase; ALT, alanine aminotransferase; ALP, alkaline phosphatase; GGT, gamma-glutamyltransferase; eGFR, estimated glomerular filtration rate; NSGP, National Glycohemoglobin Standardization Program; BTR, branched-chain amino acid ratio; APRI, AST to platelet ratio index.

**Table 2:** Univariate analyses of factors associated with the presence of sarcopenia for all cases (*n* = 372).

Variables	Sarcopenia (*n* = 56)	Non-Sarcopenia (*n* = 316)	*P* Value
Age (years)	72.5 (67.25, 76.75)	63 (52, 70)	<0.0001
Gender, male/female	27/29	144/172	0.7144
HBV/HCV/HBV and HCV/NBNC	6/31/2/17	47/193/6/70	0.4195
Body mass index (kg/m^2^)	20.6 (19.7, 22.7)	22.95 (20.7, 26)	<0.0001
Presence of LC, yes/no	29/27	118/198	0.0416
Total bilirubin (mg/dL)	0.7 (0.5, 0.95)	0.8 (0.6, 1.1)	0.0276
Serum albumin (g/dL)	3.95 (3.225, 4.3)	4.2 (3.8, 4.5)	0.0008
Prothrombin time (%)	85.3 (74.45, 98.825)	88.7 (78.45, 96.475)	0.6971
Platelet count (×10^4^/mm^3^)	15.1 (10.2, 18.6)	16.75 (11.525, 20.8)	0.2303
AST (IU/L)	32 (22, 49)	27 (21, 40.75)	0.3978
ALT (IU/L)	26 (15, 42)	23 (16, 40)	0.8374
ALP (IU/L)	287 (206, 372)	237 (192, 301)	0.0143
GGT (IU/L)	30 (18.5, 52)	28 (17, 50)	0.5899
Total cholesterol (mg/dL)	179 (142, 212)	179 (154, 205.75)	0.3933
eGFR (mL/min/1.73 m^2^)	76 (57, 92)	83 (71, 96.75)	0.0178
Serum sodium (mmol/L)	140 (138, 141)	140 (139, 142)	0.0164
HbA1c (NGSP)	5.85 (5.2, 6.1)	5.6 (5.4, 6.0)	0.4177
Serum zinc concentration (High/Intermediate/Low)	10/21/25	83/166/67	0.0009
BTR	5.19 (3.6, 7.21)	5.64 (4.34, 6.625)	0.5460
FIB-4 index	3.09 (2.15, 5.09)	2.28 (1.49, 4.01)	0.1409
APRI	0.61 (0.35, 1.42)	0.51 (0.32, 1.09)	0.8192

Data are expressed as median value (interquartile range). Abbreviations: HBV, hepatitis B virus; HCV, hepatitis C virus; NBNC, non-B and non-C; LC, liver cirrhosis; AST, aspartate aminotransferase; ALT, alanine aminotransferase; ALP, alkaline phosphatase; GGT, gamma-glutamyltransferase; eGFR, estimated glomerular filtration rate; NSGP, National Glycohemoglobin Standardization Program; BTR, branched-chain amino acid ratio.

**Table 3:** Multivariate analyses of factors linked to the presence of sarcopenia for all cases.

	Multivariate Analysis		
	Hazard Ratio	95% CI	*P* Value
Age (per one year)	1.075	1.032–1.120	0.0005
BMI (per one kg/m^2^)	0.790	0.695–0.898	0.0003
Presence of LC	1.541	0.645–3.690	0.3306
Serum albumin (per one g/dL)	0.208	0.081–0.533	0.0011
Total bilirubin (per one mg/dL)	0.439	0.176–1.093	0.0769
ALP (per one IU/L)	1.002	0.999–1.005	0.1273
eGFR (per one mL/min/1.73 m^2^)	1.001	0.985–1.018	0.8715
Serum sodium (per one mmol/L)	0.899	0.791–1.022	0.1061
Classification of serum zinc level			
Intermediate-Zn group	1.000	Reference	
High-Zn group	1.812	0.722–4.545	0.2053
Low-Zn group	3.030	1.160–7.937	0.0236

Abbreviations: BMI, body mass index; LC, liver cirrhosis; ALP, alkaline phosphatase; eGFR, estimated glomerular filtration rate; Zn, zinc; CI, confidence interval.

**Table 4:** Univariate analyses of factors associated with the presence of sarcopenia for LC cases (*n* = 147).

Variables	Sarcopenia (*n* = 29)	Non-Sarcopenia (*n* = 118)	*P* Value
Age (years)	71 (67, 76)	67 (59, 72)	0.0026
Gender, male/female	15/54	65/53	0.7448
HBV/HCV/HBV and HCV/NBNC	4/15/1/9	15/73/0/30	0.1897
Body mass index (kg/m^2^)	20.4 (19.8, 23.0)	23.05 (20.7, 26.275)	0.0010
Total bilirubin (mg/dL)	0.8 (0.6, 1.2)	1.1 (0.8, 1.625)	0.0582
Serum albumin (g/dL)	3.4 (2.95, 3.9)	3.8 (3.4, 4.125)	0.0035
Prothrombin time (%)	81.9 (58.55, 87.95)	78.25 (63.175, 88.3)	0.8211
Platelet count (×10^4^/mm^3^)	10.4 (8.05, 15.7)	10.85 (7.4, 14.525)	0.6432
AST (IU/L)	45 (29.75, 56.75)	37.5 (25, 53)	0.2028
ALT (IU/L)	32.5 (21.75, 48)	28.5 (17.75, 43.25)	0.3652
ALP (IU/L)	339.5 (234, 499.75)	264 (219, 402)	0.1226
GGT (IU/L)	41 (22, 74)	37 (21.5, 67)	0.6964
Total cholesterol (mg/dL)	144 (120.75, 188.5)	155.5 (137, 180)	0.4466
eGFR (mL/min/1.73 m^2^)	80 (66, 93.75)	82 (68, 99)	0.4683
Serum sodium (mmol/L)	139 (137, 140)	140 (138, 142)	0.0574
HbA1c (NGSP)	5.2 (5.0, 6.0)	5.6 (5.2, 6.15)	0.3641
Serum zinc classification High/Intermediate/Low	2/4/23	15/53/50	0.0016
BTR	3.7 (2.9875, 4.935)	4.245 (3.2675, 5.64)	0.1690
FIB-4 index	4.83 (3.14, 7.56)	4.27 (2.68, 6.80)	0.9641
APRI	1.29 (0.70, 1.95)	1.08 (0.60, 1.91)	0.6814

Data are expressed as median value (interquartile range). Abbreviation: HBV, hepatitis B virus; HCV, hepatitis C virus; NBNC, non-B and non-C; LC, liver cirrhosis; AST, aspartate aminotransferase; ALT, alanine aminotransferase; ALP, alkaline phosphatase; GGT, gamma-glutamyltransferase; eGFR, estimated glomerular filtration rate; NSGP, National Glycohemoglobin Standardization Program; BTR, branched-chain amino acid ratio.

**Table 5:** Multivariate analyses of factors linked to the presence of sarcopenia for LC cases.

	Multivariate Analysis		
	Hazard Ratio	95% CI	*P* Value
Age (per one year)	1.054	1.003–1.106	0.0363
BMI (per one kg/m^2^)	0.755	0.636–0.898	0.0014
Serum albumin (per one g/dL)	0.377	0.121–1.520	0.1890
Classification of serum zinc level			
Intermediate-Zn group	1.000	Reference	
High-Zn group	2.500	0.377–16.667	0.3429
Low-Zn group	6.410	1.618–25.641	0.0082

Abbreviations: BMI, body mass index; Zn, zinc; CI, confidence interval.

**Table 6:** Univariate analyses of factors associated with the presence of sarcopenia for non-LC cases (*n* = 225).

Variables	Sarcopenia (*n* = 27)	Non-Sarcopenia (*n* = 198)	*P* Value
Age (years)	73 (67, 78)	60 (48.75, 67)	<0.0001
Gender, male/female	12/15	79/119	0.6518
HBV/HCV/HBV and HCV/NBNC	2/16/1/8	32/120/6/40	0.5248
Body mass index (kg/m^2^)	20.9 (19.3, 22.3)	22.9 (20.675, 25.725)	0.0003
Total bilirubin (mg/dL)	0.6 (0.5, 0.7)	0.8 (0.6, 1.0)	0.0527
Serum albumin (g/dL)	4.2 (4.0, 4.5)	4.3 (4.1, 4.5)	0.1374
Prothrombin time (%)	93.5 (84.7, 99)	92.5 (84.95, 100.025)	0.5236
Platelet count (×10^4^/mm^3^)	17.45 (14.85, 20.7)	19.25 (15.65, 22.95)	0.5282
AST (IU/L)	23 (18, 33)	24 (19, 33)	0.3645
ALT (IU/L)	15 (12, 32)	20 (15, 35.25)	0.3151
ALP (IU/L)	243 (177, 306)	224.5 (178.75, 275.75)	0.1149
GGT (IU/L)	25 (15.75, 42.5)	23 (15, 44)	0.9164
Total cholesterol (mg/dL)	195 (165, 218)	193 (170.75, 219)	0.8202
eGFR (mL/min/1.73 m^2^)	66 (56, 92)	84 (72, 95.5)	0.0063
Serum sodium (mmol/L)	140 (138, 142)	141 (139, 142)	0.3751
HbA1c (NGSP)	6.0 (5.6, 6.4)	5.7 (5.4, 5.925)	0.0091
Serum zinc classification High/Intermediate/Low	8/17/2	68/113/17	0.8444
BTR	6.92 (5.64, 7.72)	6.25 (5.28, 7.05)	0.0544
FIB-4 index	2.13 (1.64, 2.86)	1.73 (1.13, 2.47)	0.1066
APRI	0.35 (0.28, 0.52)	0.36 (0.29, 0.63)	0.3683

Data are expressed as median value (interquartile range). Abbreviations: HBV, hepatitis B virus; HCV, hepatitis C virus; NBNC, non-B and non-C; LC, liver cirrhosis; AST, aspartate aminotransferase; ALT, alanine aminotransferase; ALP, alkaline phosphatase; GGT, gamma-glutamyltransferase; eGFR, estimated glomerular filtration rate; NSGP, National Glycohemoglobin Standardization Program; BTR, branched-chain amino acid ratio.

**Table 7:** Multivariate analyses of factors linked to the presence of sarcopenia for non-LC cases.

	Multivariate Analysis		
	Hazard Ratio	95% CI	*P* Value
Age (per one year)	1.071	1.004–1.142	0.0249
BMI (per one kg/m^2^)	0.746	0.602–0.923	0.0021
eGFR (per one mL/min/1.73 m^2^)	0.985	0.955–1.015	0.3257
HbA1c (per one)	1.280	0.727–5.376	0.2127

Abbreviations: BMI, body mass index; eGFR, estimated glomerular filtration rate; CI, confidence interval.

**Table 8:** Relationship between serum zinc concentration and baseline characteristics.

	*r*	*P* Value
Age	−0.186	0.0003
Body mass index	−0.032	0.5424
Total bilirubin	−0.355	<0.0001
Serum albumin	0.654	<0.0001
Prothrombin time	0.395	<0.0001
Platelets	0.295	<0.0001
Serum sodium	0.249	0.8923
eGFR	−0.020	0.7029
Total cholesterol	0.266	<0.0001
AST	−0.230	<0.0001
ALT	−0.065	0.2126
ALP	−0.245	<0.0001
GGT	−0.206	<0.0001
HbA1c (NSGP)	0.133	0.0242
BTR	0.496	<0.0001
FIB-4 index	−0.360	<0.0001
APRI	−0.289	<0.0001

Abbreviations: eGFR, estimated glomerular filtration rate; AST, aspartate aminotransferase; ALT, alanine aminotransferase; ALP, alkaline phosphatase; GGT, gamma-glutamyltransferase; NSGP, National Glycohemoglobin Standardization Program; BTR, branched-chain amino acid ratio; APRI, AST to platelet ratio index.
